# Prevalence and molecular characterization of multidrug-resistant *M*. *tuberculosis* in Jiangxi province, China

**DOI:** 10.1038/s41598-019-43547-2

**Published:** 2019-05-13

**Authors:** Dong Luo, Qiang Chen, Guangchu Xiong, Yiping Peng, Tao Liu, Xiaowen Chen, Lingbing Zeng, Kaisen Chen

**Affiliations:** 1 Department of Clinical Laboratory, the First Affiliated Hospital of Nanchang University, Nanchang, 330006 China; 2 Department of Clinical Laboratory, Jiangxi Provincial Chest Hospital, Nanchang, 330006 China

**Keywords:** Molecular medicine, Diseases, Tuberculosis

## Abstract

Multidrug-resistant *Mycobacterium tuberculosis* (MDR-TB) is a severe health threat to human beings; however, the epidemic and molecular characteristics exist along with the change in the geographic environment and genealogy. Jiangxi province is located in southeast China, which is a high-MDR-TB burden area. Rifampin (RIF) and isoniazid (INH) are the most important first-line anti-tuberculosis drugs. The major drug target genes include *rpoB* for RIF and *katG*, *inhA*, and *ahpC* for INH. To determine the frequency and distribution of mycobacterial mutations in these genes, we sequenced specific genes of *M*. *tuberculosis* that are associated with resistance to RIF and INH in 157 phenotypic MDR isolates. At the same time, RD105 DTM-PCR and 15 loci MIRU-VNTR were performed to demonstrate the genetic lineage. It was shown that the Beijing genotype was predominant (84.1%) among these strains. The results also showed mutations within the 81 bp core region of *rpoB* in 93.6% of strains and mutations in a structural gene (*katG*) and two regulatory regions (the promoter of *inhA* and intergenic region of *oxyR-ahpC*) were shown in 88.5% of phenotypic MDR isolates. There were no significant differences in codon mutations between the Beijing and non-Beijing genotypes, as well as the clustered and no-clustered strains. The most prevalent mutations involved in RIF and INH were Ser531Leu in *rpoB* (55.4%) and Ser315Thr in *KatG* (56.1%), respectively. There was no significant difference in RIF and INH resistance between MDR-TB and other drug-resistant tuberculosis (DR-TB). The results demonstrated that some MDR-TB patients are predicted to have recent transmission.

## Introduction

Tuberculosis (TB) is a global public health threat. The World Health Organization (WHO) has estimated 10.4 million new TB cases in 2016, including 0.49 million multidrug-resistant tuberculosis (MDR-TB) cases^1^. The countries with the largest number of MDR include China, India, and the Russian Federation^1^. The basis for MDR-TB includes delayed diagnosis, prolonged or ineffective treatment, and person-to-person recent transmission. China is 1 of the 27 high MDR-TB burden countries. The prevalence of MDR-TB in China was 5.3% in recent years^2^. Although concentrated efforts to control TB has resulted in a sharp decrease in the incidence of TB in recent years, China still had the largest number of MDR cases worldwide in 2016^1^.

Jiangxi province is located in southeast China. Jiangxi province is a resource-limited, high TB burden area with approximately 45.0 million inhabitants according to the current estimated population in China^3^. The prevalence of TB in China in 2010 was 463/100,000 among the general population, which is far higher than the average prevalence of TB in countries worldwide. Yuan and colleagues reported that the prevalence of MDR-TB was 19.8% based on hospital data, which is nearly the highest rate reported in China^4^. Because of small sample sizes in previous studies, further information is required for effective management of MDR-TB patients^5^.

To determine the prevalence and pattern of mutations, drug target genes for rifampin (RIF) and isoniazid (INH) in these MDR-TB strains and other drug-resistant tuberculosis (DR-TB) strains have been sequenced and detected. The genetic loci were the 81 bp core region of *rpoB* in RIF and *KatG*, the promoter region of *inhA*, and *oxyR-ahpC* in INH^6,7^. Furthermore, RD105 deletion-targeted multiplex PCR (DTM-PCR) and mycobacterial interspersed repetitive unit-variable number of tandem repeats (MIRU-VNTR) genotyping were performed to allow a better understanding of MDR-TB genetic lineage and mutant patterns.

## Results

### Study population and MDR-TB strains isolated

**Figure 1:**
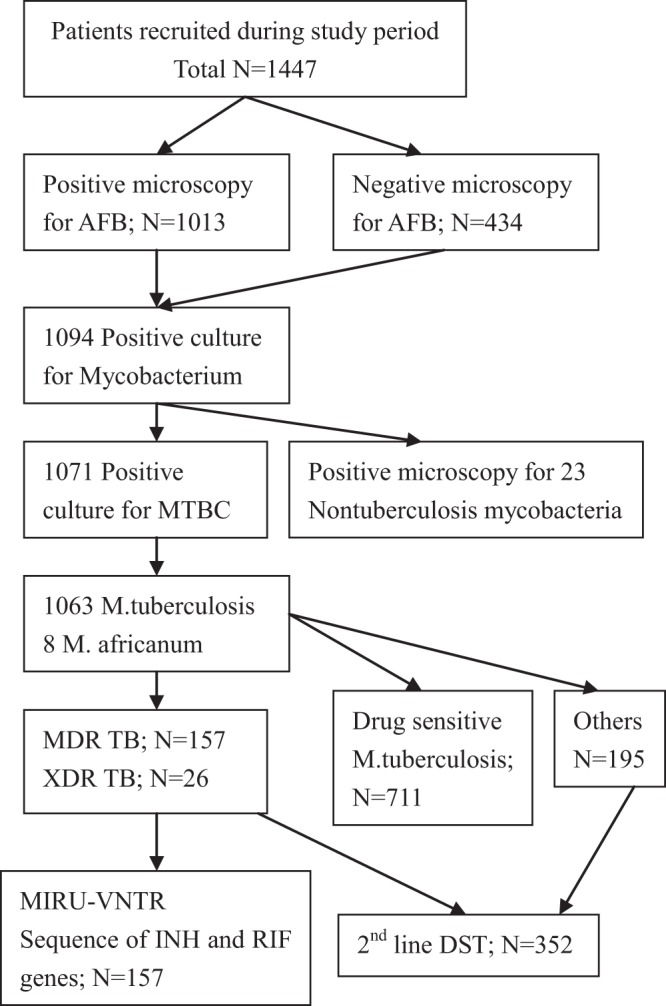
Study profile of MDR TB patients at Jiang.

Of the 1447 samples collected, 1013 were smear-positive, 1094 had culture results, and 1071 were available for analysis. There were 157 MDR-TB patients and 26 were identified as XDR strains by second-line DST. One hundred ninety-five other DR-TB and 711 drug-susceptible TB (DS-TB) cases were included in this investigation (Fig. 1). Furthermore, 53 patients were primary drug-resistant (including 10 XDR-TB patients) and 104 were acquired drug-resistant (including 16 XDR-TB patients). The detailed results are shown in Table 1.

**Table 1:** Characteristics of MDR TB patients from Jiangxi Province, China.

Variable	MDR TB N = 157	DS TB N = 711	χ^2^	P value
Tuberculosis category			261.96	0.001
New	53	637		
Recurrent	63	61		
Treatment failure	41	13		
Age, years			20.74	0.001
≤30	63	165		
30–50	50	247		
≥50	44	299		
Marital status			0.65	0.42
Unmarried	23	123		
Married	134	588		
Sex			1.44	0.23
Male	103	501		
Female	54	210		
HIV status			0.66	0.42
Positive	5	15		
Negative	152	696		
Smoking			52.42	0.001
Never	31	223		
Previous	58	367		
Current	68	121		
Sputum smear			0.04	0.84
Negative	22	104		
Positive	135	607		
Cavity			15.76	0.001
Yes	28	54		
No	129	657		
Profession			32.35	0.001
Farmer	91	239		
No farmer	66	472		

### Clinical characteristics of MDR-TB patients

We compared MDR-TB (n = 157) and DS TB patients (n = 711) with respect to clinical characteristics (Table 1). One hundred thirteen of 157 MDR-TB patients (71.0%) were <50 years of age, and 412 of 711 DS-TB patients (57.9%) were <50 years of age. For a more detailed profile, we also compared MDR-TB with other DR-TB patients (n = 195). The results are shown in Table 2, and included 127 single drug-resistant TB, 123 two drug-resistant TB, 98 three drug-resistant TB, and 4 four drug-resistant TB patients. Moreover, 20 patients were HIV-positive. Ninety-one of 157 MDR-TB patients (58.0%) MDR-TB patients, 94 of 195 DR TB patients (48.2%), and 239 of 711 DS-TB patients (33.6%) were farmers. MDR-TB patients (28/157 [17.8%]) were more likely to present with lung cavitations compared with DS-TB patients (54/711 [7.6%]). In addition, current smoking was more severe in MDR-TB patients (68/157 [43.3%]) compared with DS-TB patients (121/711 [17.0%]; χ^2^ = 29.65, *P* = 0.001). There were no significant differences between MDR-TB and DS-TB patients with respect to marital status, gender, HIV status, and sputum smears.

**Table 2:** Profiles of 352 Drug-resistant TB in Jiangxi Province, China.

Drug	Strains (n = 294)
Resistant single drug	
RIF	28
INH	60
EMB	2
SM	37
Resistant two drugs	
RIF + INH	58
RIF + SM	18
INH + SM	38
RIF + EMB	8
INH + EMB	1
Resistant three drugs	
RIF + INH + EMB	88
RIF + INH + SM	7
INH + SM + EMB	3
Resistant four drugs	
RIF + INH + SM + EMB	4

### Molecular characterization of INH and RIF in DR-TB isolates

Because most RIF-resistant strains harbor mutations within the 81-bp RIF-resistant determining region (RRDR), the 442-bp fragment of *rpoB* (including the 81-bp core region) was detected. Altogether, 94.3% (147/157) of the MDR isolates harbored mutations in 12 codons of RRDR, while 10 of the RIF-resistant strains lacked this mutation. The most frequently mutated *rpoB* was codon 531 (90/157 [57.3%]), including 87 Ser531Leu and 3 Ser531Phe. The second most frequently mutated point was codon 526, including 2 His526Asp, 6 His526Tyr, 10 His526Pro, 3 His526Leu, and 5 His526Asn. All 12 double-mutant strains had mutations in the core region, but no mutations existed outside of the RRDR. There were two nonsense mutations (TTC505TTT), and no strains had triple mutations (Table 3). To elucidate the isoniazid-resistant pattern, we sequenced gene fragments of *katG*, the promoter region of *inhA*, and the intergenic region of *oxyR-ahpC*. Of the MDR-TB strains, 89.3% (140/157) had one or more mutations in these regions (Table 4). Of the 140 mutants, the most frequent mutation site was *katG*315 (108/157 [68.8%]), and 64.3% (90/140 strains) had single *katG* mutations, including 87 Ser315Thr and 2 Ser315Asn. Furthermore, 19.7% (31/157) of the MDR-TB isolates harbored mutations in the *inhA* region. Seventeen of the 31 isolates had no additional mutations, 14 had one additional mutation in *katG*315, and 9 had additional mutations in the intergenic region of *oxyR*-*ahpC*. Among157 MDR-TB isolates, 12.1% (17/157) of the isolates had nucleotide substitutions in the intergenic region of *oxyR-ahpC* (Table 4). In addition, we also identified some novel types of mutations, such as the *rpoB* (ATG515GTG and CGA529CTG, *KatG* (ACG322GCG and ACG322ACC), and *inhA* genes (CGA *inhA*-3 CTG; G → P and ATG *inhA*-3 GTG; G → G) based on the TB DR database.

**Table 3:** Distribution of mutations of rpoB gene among 157 MDR TB isolates in Jiangxi Province, China.

Locus	Change		No. (%) of isolates
	Nucleotide	Amino acid	
rpoB531	TCG → TTG	Ser → Leu	87 (55.4)
	TCG → TTT	Ser → Phe	3 (1.9)
rpoB526	CAC → GAC	His → Asp	2 (1.3)
	CAC → TAC	His → Tyr	1 (0.6)
	CAC → CCC	His → Pro	13 (8.3)
	CAC → CTC	His → Leu	3 (1.9)
	CAC → AAC	His → Asn	1 (0.6)
rpoB526/533	CAC → AAC	His → Asn	1 (0.6)
	CTG → CCG	Leu → Pro	
rpoB526/529	CAC → AAC	His → Asn	5 (3.2)
	CGA → CTG	Arg → Leu	
rpoB516	GAC → GTC	Asp → Val	10 (6.6)
rpoB516/511	GAC → GGC	Asp → Gly	1 (0.6)
	CTG → CCG	Leu → Pro	
rpoB533	CTG → CCG	Leu → Pro	6 (3.8)
rpoB533/515	CTG → CCG	Leu → Pro	1 (0.6)
	ATG → GTG	Met → Val	
rpoB533/518	CTG → CCG	Leu → Pro	3 (1.9)
	AAC → ACC	Asn → Tyr	
rpoB513	CAA → AAA	Gln → Lys	3 (1.9)
rpoB513/511	CAA → AAA	Gln → Lys	1 (0.6)
	CTG → CCG	Gln → Lys	
rpoB505	TTC → TTT	Phe → Phe	2 (1.3)
rpoB522	TCG → TTG	Ser → Trp	1 (0.8)
rpoB519	AAC → AAG	Asn → Lys	3 (1.9)
Wild type	None	None	10 (6.4)

**Table 4:** Distribution of mutations in KatG and two regulatory regions (the promoter of mabA-inhA and oxyR-ahpC intergenic region among 157 MDR TB isolates in Jiangxi Province, China.

Locus	Change		No. (%) of isolates
	Nucleotide	Amino acid	
KatG315	AGC → ACC	Ser → Thr	88 (56.1)
	ACG → AAC	Ser → Asn	2 (1.3)
KatG322	ACG → GCG	Thr → Ala	5 (3.2)
	ACG → AAC	Thr → Asn	2 (1.3)
KatG315/inhA-15	AGC → ACC	Ser → Thr	7 (4.5)
	C → T	None	
KatG315/ahpC-15	AGC → ACC	Ser → Thr	2 (1.3)
	C → T	None	
KatG315/ahpC-52	AGC → ACC	Ser → Thr	1 (0.6)
	C → G	None	
KatG315/ahpC-6	AGC → GCG	Ser → Ala	1 (0.6)
	G → T	None	
KatG315/inhA-3	AGC → ACC	Ser → Thr	2 (1.3)
	GGA → CCG	Gly → Pro	
KatG315/inhA-3/ahpC-39	AGC → ACC	Ser → Thr	5 (3.2)
	GGA → GGG	Gly → Gly	
	C → G	None	
inhA-15	C → T	None	17 (10.8)
ahpC-39	C → A	None	4 (2.5)
ahpC-10	C → T	None	2 (1.3)
ahpC-9	G → T	None	1 (0.6)
ahpC-32	G → A	None	1 (0.6)
Wild type	None	None	17 (10.8)

### Genotyping of MDR-TB strains

One hundred thirty-two of 157 (84.1%) isolates were shown to be the Beijing genotype using the DTM-PCR method. Furthermore, 25 non-Beijing genotype strains were considered as various lineages using a similarity search on the MIRU-VNTR*plus* website. The most prevalent genotype among the non-Beijing genotype strains was the S family (8/25 [32.0%]), followed by Cameroon (3/25 [12.0%]), and NEW-1 (2/25 [8.0%]). The remaining 12 of 25 strains (48.0%) could not be assigned to a single lineage using this genotyping method. A dendrogram based on the 15-loci MIRU-VNTR method was constructed using the UPGMA method (Additional File 1). Altogether, MIRU-VNTR cluster analysis showed that the 157 strains were classified into 114 genotypes. A total of 105 strains had unique patterns, while the remaining 52 strains were grouped into 13 clusters. The largest cluster had 14 strains and 6 clusters had 2 strains, resulting in a clustering rate of 33.1% (52/157) and a recent transmission rate of 24.8% (39/157).

### Relationship between genotypes, clustering, and drug resistance

The 15-loci MIRU-VNTR and DTM-PCR analysis showed 118 different genotypes and 132 Beijing genotype strains in the 157 MDR-TB isolates. A dendogram (Additional File 1) showed 105 of 157 (66.9%) singletons and 13 clusters confirmed by 157 isolates. Thirteen clusters, including 52 isolates, were identified as 50 Beijing family isolates and 2 non-Beijing family isolates. Beijing family isolates had no significant clustering ability (*P* = 0.057; OR: 3.16; 95% CI: 0.91–10.92) compared with non-Beijing family isolates.

To understand the relationship of mutations in recent transmission, we compared the mutated patterns in clustered and non-clustered strains. The results showed that there was no significant difference in all mutated codons, such as *rpoB*531 and *KatG*315, which were the most frequent mutant codons in RIF and INH in this study (Additional Table 1).

## Discussion

Based on our hospital-based investigation of TB cases, the isolation rate for MDR-TB was 14.7% (157/1071) during the study period in Jiangxi province, China, which was slightly lower than previously reported^4^. The Jiangxi province Chest Hospital is the only TB tertiary hospital and almost all local severe TB patients (such as recurrent and treatment failure) were collected for further treatment. As a result, the MDR-TB isolation rate could be higher than the average level in Jiangxi province; however, these patients come from all places. The value of our study is the molecular characterization and prevalence of MDR-TB in the Jiangxi region.

In the present study, only one-half of the cases were new (33.8% [53/157]); the other cases were previously treated or recurrent patients (66.2% [104/157]), suggesting that the strict Directly Observed Treatment Short Course (DOTS) was not performed completely, including a lack of professional personnel, insufficient support, and poor patient adherence to DOTS. Thus, DOTS must be performed strictly to prevent and cure MDR-TB patients. We found that farming is an important risk factor for MDR patients, which was in agreement with results from other studies^8,9^. A possible reason for this observation is that most farmers have to work far away from their home town and have poor adherence to treatment due to heavy workloads or irregular lives. Smoking is another risk factor for MDR patients because smoking can decrease local lung immunity, thus leading to easier transmission and worse therapeutic effects^10,11^.

Moreover, this study provided a detailed genetic analysis of 157 MDR-TB isolates using DTM-PCR and the 15 loci MIRU-VNTR method. Our data revealed genetic linkage by genetic lineage identification and cluster analysis, suggesting that these strains were important epidemically, and relative and recent transmission possibly occurred. Of 157 MDR patients, 52 clustered cases and 105 unique cases were acquired using established methods. Because HGDI was very high (Additional Table 2), we confirmed that the 15 loci MIRU-VNTR method was suitable for typing these isolates. For example, among 52 clustered patients, 16 were identified with epidemiologic links. Acquired drug-resistance of *M*. *tuberculosis* relies on the selective pressure of treatment failure, such as inappropriate chemotherapy, poor adherence to treatment, or inadequate monitoring; however, transmission is also considered to be an important role as the MDR-TB source^12^. As shown by the results, there were a total of 13 clusters, including 12 Beijing family clusters and 1 non-Beijing family cluster. Although some studies have reported that Beijing family strains are more prone to transmission^13,14^, the results herein did not confirm this finding, possibly because the quantity of specimens was too low. Of note, the Beijing genotype is still the predominant genotype throughout Jiangxi^5^, accounting for 84.1% in the present study, which is in agreement with the findings from most geographic areas in China^15^.

The detection of mutations in the 81-bp core region of rpoB was important in predicting phenotypic RIF-resistance. Some studies have shown that mutations within the RRDR could acquire >95% of RIF-resistant isolates^16^. In the current study, there were 197 strains with mutations in the RRDR in all RIF-resistant isolates (211 strains), including 147 MDR TB and 50 other resistant strains. At the same time, there were 259 INH-resistant strains, including 157 MDR-TB and 102 other resistant strains. The most important locus among the resistant strains was *KatG*315. RIF resistance was usually accompanied with resistance to INH. Therefore, RIF resistance was an extremely sensitive marker of MDR-TB in some places^17^. In the current study, 93.4% of the isolates had mutations in the 81-bp core region of *rpoB*, which was similar to results reported in Shanghai^16,17^, but higher than the results reported from Hunan, China. Our results also showed that the most frequent mutation was at *rpoB*531, *rpoB*526, *rpoB*516, *rpoB*533, and *rpoB*513 in the RRDR among the MDR-TB isolates and there were no obvious differences between MDR-TB and other RIF-resistant strains. Although the frequencies of mutations may differ, the results were consistent with most places in the world^18,19^.

Of 157 MDR-TB isolates, 74.5% (117/157 strains) of INH resistance-conferring mutations were most frequently detected at the *KatG* gene, which was similar to other INH-resistant strains. At the same time, 68.8% of strains (108/157) had *KatG*315 mutations in all MDR-TB strains and 83.3% (90/108) of *KatG*315 in Beijing genotype strains. This mutation had different rates in different places. A study found that 100% of MDR *katG* mutations were at codon 315 in central China^20^. In contrast, *KatG*315 mutations accounted for INH resistance of 50.6% in Mexico and 53.7% in Argentina^21,22^. Discrepancies can be attributed to regional variations. Our study found 21.7% (34/157 isolates) of mutations in the promoter region of *inhA* of the MDR samples and 47.1% (16/34) had *inhA* regional mutations without a mutation in *katG*. The *inhA*-15 mutation was most frequent in the promoter region, and the second most frequent mutation conferring INH resistance in our study, which was similar to Argentina^23^. We found 12.1% (19/157) of mutations in *oxyR-ahpC* of the MDR strains and a single mutation in the intergenic region (only 3.8% [6/157]). Although some studies have reported that there are rare mutations in the intergenic domain of *oxyR-ahpC* among INH-resistant strains, mutations in the intergenic region result in the up-regulation of *ahpC*, which has been associated with deficiency of catalase-peroxidase activity and might be valuable for mycobacteria survival^24^.

We had repeat DNA sequencing and DST on L-J slope medium for mismatch results on phenotypic and genetic resistance tests (18 for INH and 10 for RIF) of the MDR-TB and found there were no mistakes. As a result, there may be an additional mechanism involved in the INH and RIF resistance of these isolates, and possibly the mutations in other structural genes or other loci.

In summary, our results indicated that the most frequent mutations in the *rpoB*, *katG*, *inhA*, and *ahpC* were *rpoB*531, *katG*315, *inhA*-15, and *ahpC*, which is consistent with those from some regions in the world^19^. Although there were some novel mutations in the *rpoB*, *katG*, or *inhA* genes, these isolates also had accompanying mutations in *rpoB* (including 526 or 533 points) or *KatG*315. More studies need to be performed to determine if these mutations are associated with drug resistance. There were no significant differences between MDR-TB and other DR-TB strains. Beijing family strains had no significant differences from non-Beijing family strains in codon mutations, including any mutant loci. MDR-TB might originate from DR-TB mutations for non-standard treatment or other reasons, as well as recent transmission. Moreover, our studies can also offer valuable information for designing diagnostic tests to detect RIF and INH resistance of MDR-TB in the local place.

## Material and Methods

### Ethics statement

The study was approved by the Ethics Committee of the First Affiliated Hospital of Nanchang University (Nanchang, China [approval number: 2014036]). All patients had informed consent for study participation, and all personal data were kept confidential. At the same time, we confirmed that all methods were performed in accordance with the relevant guidelines and regulations in China.

### M. tuberculosis isolates

This survey was conducted from January 2014 to December 2016 at the Jiangxi Chest Hospital located in Nanchang city, the capital of Jiangxi province. The legal entity is the unique tertiary care TB hospital in the Jiangxi area, serving TB patients within the region. A total of 1447 pulmonary tuberculosis patients were recruited during this study period. In addition, three sputum samples at different time points (spot, early, and night) were collected from each patient for acid-fast bacilli (AFB) using the Ziehl Neelsen (ZN) method and Löwenstein-Jensen culture. Biochemical methods, including the catalase test, nitrate reduction, thiophene carboxylic acid hydrazide, p-nitrobenzoic acid, and smooth appearance of colonies, were used to differentiate *Mycobacterium tuberculosis* complex (MTBC) and non-tuberculosis mycobacteria (NTM).

### Drug susceptibility testing

For all *M*. *tuberculosis* isolates collected, first-line drug susceptibility testing (DST) was routinely performed on Löwenstein-Jensen (L-J) medium using the proportion method. To further offer therapeutic options, DST for four representative second-line drugs (ofloxacin, kanamycin, amikacin, and capreomycin) was performed for any first-line drug-resistant strains in the study. Those tests were performed using the following drug concentrations: INH (0.2 μg/ml); RIF (40.0 μg/ml); streptomycin ([SM] 4.0 μg/ml); ethambutol ([EMB] 2.0 μg/ml); ofloxacin (2.0 μg/ml); kanamycin ([KAN] 30.0 μg/ml); amikacin ([AMK] 40.0 μg/ml); and capreomycin ([CAP] 40.0 μg/ml). Quality control was routinely performed during DST using the reference strain (H37Rv [ATCC27294]).

### Data collection and definitions

During the survey period, all information on pulmonary TB culture-positive patients (n = 1071) at the Jiangxi Chest Hospital was enrolled in the study. The following clinical information was obtained from patients’ medical records: gender; age; marital status; TB treatment history; presence of cavitations on chest radiograph; HIV status; smoking; sputum smears; and careers. MDR-TB was defined as a strain showing resistance to at least INH and RIF, while XDR-TB was defined as MDR-TB with additional resistance to ofloxacin and at least one of the following three second-line injectable drugs: kAN; AMK; or CAP. DR-TB was defined as a strain showing resistance to at least RIF or INH. DS-TB was defined as a strain showing pan-susceptible to all first-line anti-tuberculosis drugs. New or recurrence or failure treatment TB case was defined as previously described^25^.

### DNA extraction and sequencing

Bacterial genomic DNA was obtained from isolates by the boiled lysis method^26^. All strains resistance to RIF and/or INH were included in this study. The following fragments were amplified and sequenced: 442 bp of the *rpoB* gene (including the 81-bp core region); 518 bp of the *katG* gene (including codons 315); 248 bp of the *inhA* promoter region; and 359 bp of the intergenic region of *oxyR-ahpC* (454-FLX sequencer (Sangon Bio Co., Shanghai, China). Primer sequences and PCR conditions were based on previously reported studies^27,28^. Primers were synthesized by Sangon Bio Co. The PCR mixtures were prepared using 2 × *Taq* MasterMix (Tiangen Co., Beijing, China). Sequencing data was assembled and analyzed by Mega5.04 software.

### Genotyping method

Fifteen loci MIRU-VNTR genotyping was performed to genotype these MDR-TB isolates by following the protocol described by Chen *et al*.^3^. In brief, all VNTR were amplified by PCR according to literature reported^29^, then PCR products were observed by electrophoresis on 1.5% agarose gels, and the size analysis of PCR fragments was performed using the Gel Image Analysis System (LiuYi Co., Beijing, China). All MDR-TB strains were identified whether the Beijing genotypes or not using the RD105 DTM-PCR method^30^. The number of repetitions of various MIRU-VNTR loci was determined by comparison with the reference strain H37Rv.

### Data management and analysis

Clustering analysis was done using the unweighted pair group method with arithmetic average (UPGMA). Discrimination of the locus combination was calculated using the HDGI^31^:

$$HGI=1-\frac{1}{N(N-1)}\sum _{j=1}^{S}{\rm{n}}j(nj-1)$$

where N is the total number of isolates in the typing method, s is the number of distinct patterns discriminated by MIRU-VNTR, and nj is the number of isolates belonging to the jth pattern. Allelic diversity (h) was done using equation:

$${\rm{h}}=1-\sum {{\rm{xi}}}^{2}[{\rm{n}}/({\rm{n}}-1)]$$

where n is the number of isolates and xi the frequency of the ith allele at the locus. Where *x*_i_ is the frequency of the *ith* allele at the locus^32^. The clustering rate was defined as (nc-c)/n, whereby nc is the total number of clustered cases, c is the number of clusters, and n is the total number of isolates^23^. Heterogeneity of sex, age, or others in MDR-TB and DS-TB was assessed using the chi-square test using SPSS17.0 (SPSS Inc., Chicago, IL, USA). Values of *P* < 0.05 were considered statistically significant.

## Supplementary information

Supplementary information

## Data Availability

All patient information and sequencing data of this study are available from the corresponding authors upon request.
