# Supplementary material for: Prevalence and molecular characterization of multidrug-resistant M. tuberculosis in Jiangxi province, China
Source: Sci Rep. 2019 May 13;9:7315. doi: 10.1038/s41598-019-43547-2 (PMC6513856; doi:10.1038/s41598-019-43547-2)
Supplement: Supplementary file 1 — Supplementary information [file 41598_2019_43547_MOESM1_ESM.pdf]

Prevalence and molecular characterization of multidrug resistant *M.tuberculosis* in Jiangxi province, China

Luo dong[1], Chen qiang[2], Xiong guangchu[3], Peng yiping[4], Liu tao[5], Chen xiaowen[6], Zeng lingbing[7], Chen kaisen[8*]

1. Luo dong. Department of clinical laboratory, the first affiliated hospital of Nanchang university, Nanchang, China; 330006; luodong1993@foxmail.com

2. Chen qiang. Department of clinical laboratory, the first affiliated hospital of Nanchang 3. university, Nanchang, China; 330006; sb674389899@qq.com

3. Xiong guangchu. Department of clinical laboratory, Jiangxi provincial chest hospital, Nanchang, China, 330006; xionggc100@126.com

4. Peng yiping. Department of clinical laboratory, Jiangxi provincial chest hospital, Nanchang, China, 330006; pyp74@126.com

5. Liu tao. Department of clinical laboratory, the first affiliated hospital of Nanchang university, Nanchang, China; 330006; 304950764@qq.com

6. Chen xiaowen. Department of clinical laboratory, the first affiliated hospital of Nanchang university, Nanchang, China; 330006; 13767045805@qq.com

7. Zeng lingbing. Department of clinical laboratory, the first affiliated hospital of Nanchang university, Nanchang, China; 330006; lingbing_zeng@163.com

8. Chen kaisen. Department of clinical laboratory, the first affiliated hospital of Nanchang university, Nanchang, China; 330006; chenks100@126.com. (+86) 018970968159; Fax: (+86) 0791 88692594.

*: Correspondence author: Kaisen Chen, Department of Clinical Laboratory, First Affiliated Hospital of NanChang University, 17 Yongwaizhengjie, Nanchang, 330006, China. Tel: (+86) 18970968159. E-mail: Chenks100@126.com

Additional file 1 UPGMA dendrogram of 157 MDR TB isolates in this study

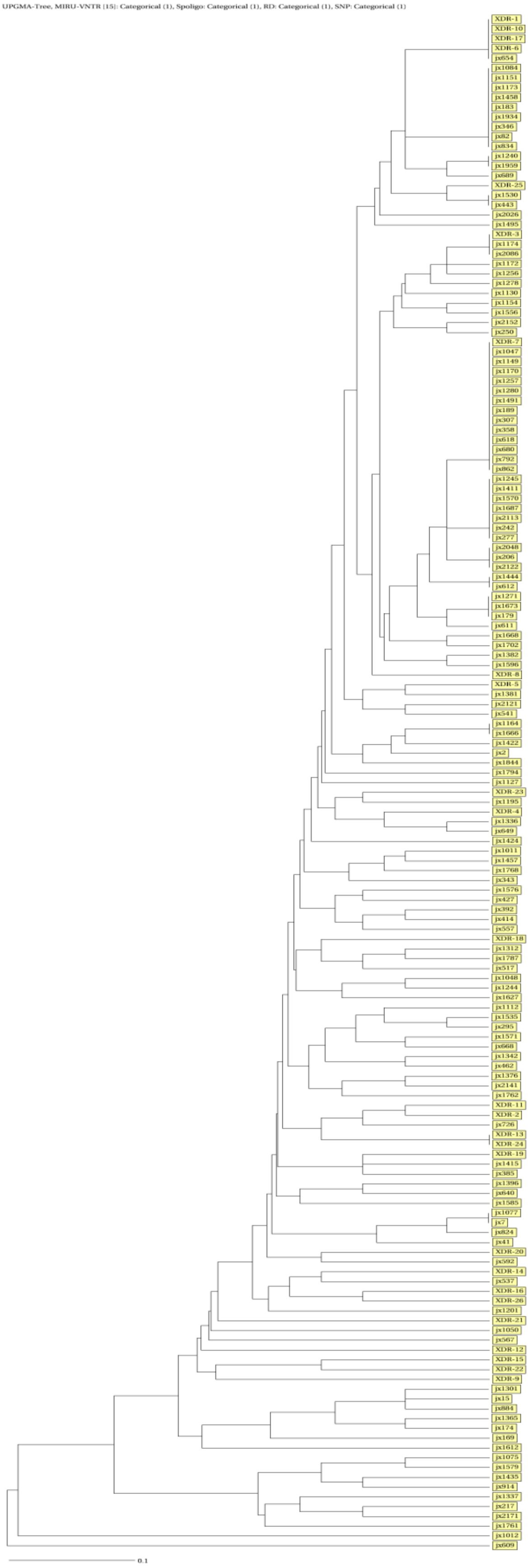

Additional table 1 Relation between mutation patterns and clusters or Beijing genotype strains of MDR TB

Mutation	Clustered No. (%)44	Non-clustered No. (%)	OR (95% CI)	P value	Beijing genotypes No. (%)	Non-Beijing genotypes No. (%)	OR (95% CI)	P value
rpoB 531	24	64	1.038<br>0.579-1.862	0.900	76	14	0.642<br>(0.324-1.271)	0.201
rpoB 526	6	20	1.298<br>0.489-3.446	0.600	20	6	1.046<br>(0.390-2.805)	0.929
rpoB 516	4	7	0.681<br>0.190-2.443	0.554	11	0	Could not calculate	_
rpoB 533	3	7	0.909<br>(0.225-3.672)	0.893	9	1	0.387<br>0.047-3.162	0.359
rpoB513	1	3	1.168<br>(0.118-11.533)	0.894	4	0	Could not calculate	_
Kat315	33	76	0.897<br>(0.524-1.534)	0.691	90	18	0.689<br>(0.367-1.295)	0.246
inhA	12	17	0.552<br>(0.244-1.249)	0.150	24	5	0.726<br>(0.258-2.043)	0.543
ahpC	5	14	1.090<br>(0.371-3.207)	0.875	17	4	0.820<br>(0.259-2.596)	0.736

Additional table 2 Allelic diversity of different MIRU-VNTR markers for the whole samples and for the Beijing genotype isolates

VNTR Alias	Repeats number	Allelic diversity(h)	Repeat number	Allelic diversity(h)
	All isolates (N=157)		Beijing isolates (N=132)	
Mtub04	1(8); 2(38); 3(89); 4(22)	0.6027	1(5); 2(31); 3(76); 4(20)	0.5935
ETRC	2(2);3(3); 4(152)	0.0626	2(2); 3(2); 4(128)	0.0597
ETRD	1(3); 2(1); 3(150); 4(3)	0.0870	1(3); 2(1); 3(126); 4(2)	0.0888
MIRU40	1(3); 2(20); 3(128); 4(3); 5(3)	0.3496	1(1); 2(10); 3(117); 4(2); 5(2)	0.2097
MIRU10	1(8); 2(26); 3(119); 4(3); 5(1)	0.3976	1(8); 2(13); 3(105); 4(6)	0.3544
MIRU16	1(2); 2(7); 3(126); 4(12)	0.3498	1(1); 2(5); 3(116); 4(11)	0.2210
Mtub21	2(6); 3(21); 4(119); 5(8); 6(3)	0.4058	2(1); 3(11); 4(113); 5(4); 6(3)	0.2607
Qub11b	1(4); 2(2); 3(2); 4(134); 5(13); 7(2)	0.2652	1(2); 2(1); 3(1); 4(115); 5(10); 7(2)	0.2364
ETRA	2(3); 3(27); 4(125); 5(1)	0.3382	2(1); 3(15); 4(115)	0.2297
Mtub30	2(13); 3(1); 4(142)	0.1761	2(5);3(1); 4(125)	0.1025
MIRU26	1(3); 2(1); 3(1); 4(11); 5(19); 6(22); 7(88); 8(9); 9(2)	0.6468	2(1); 4(7); 5(9); 6(17); 7(86); 8(9); 9(2)	0.5507
ETRE	2(5); 3(8); 4(19); 5(108); 6(13); 7(1)	0.5047	2(3); 3(2); 4(18); 5(85); 6(8); 7(1)	0.5657
Mtub39	3(4); 4(131); 5(14); 6(8)	0.2944	3(3); 4(112); 5(12); 6(5)	0.2719
Qub26	2(5); 3(4); 4(5);5(89); 6(3); 7(1); 8(49)	0.5818	2(2); 3(4); 4(4);5(69); 6(3); 7(1); 8(49)	0.5908
Qub4156	0(1); 2(23); 3(128); 5(2); 7(3)	0.3019	2(12); 3(119); 5(2)	0.1802
HGDI		0.9859		0.9803
